# Low-buoyancy thermochemical plumes resolve controversy of classical mantle plume concept

**DOI:** 10.1038/ncomms7960

**Published:** 2015-04-24

**Authors:** Juliane Dannberg, Stephan V. Sobolev

**Affiliations:** 1 Helmholtz Centre Potsdam, GFZ German Research Centre for Geosciences, Telegrafenberg, 14473 Potsdam, Germany; 2 Institute of Earth and Environmental Science, University of Potsdam, Karl-Liebknecht-Straße 24-25, 14476 Potsdam-Golm, Germany

## Abstract

The Earth's biggest magmatic events are believed to originate from massive melting when hot mantle plumes rising from the lowermost mantle reach the base of the lithosphere. Classical models predict large plume heads that cause kilometre-scale surface uplift, and narrow (100 km radius) plume tails that remain in the mantle after the plume head spreads below the lithosphere. However, in many cases, such uplifts and narrow plume tails are not observed. Here using numerical models, we show that the issue can be resolved if major mantle plumes contain up to 15–20% of recycled oceanic crust in a form of dense eclogite, which drastically decreases their buoyancy and makes it depth dependent. We demonstrate that, despite their low buoyancy, large enough thermochemical plumes can rise through the whole mantle causing only negligible surface uplift. Their tails are bulky (>200 km radius) and remain in the upper mantle for 100 millions of years.

## Abstract (web summary)

The classic mantle plume concept explains large igneous provinces and hotspot magmatism, but often contradicts observed surface uplift and plume morphology. Here, the authors present a plume model that better supports observations by considering low-buoyancy plumes containing up to 15% of recycled oceanic crust.

## Graphical abstract

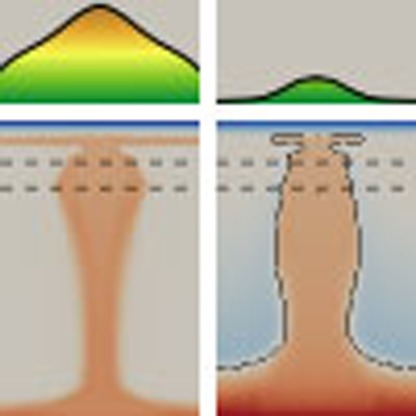

Classical starting mantle plume models predict large plume heads that cause kilometre-scale surface uplift, followed by the eruption of a large igneous province (LIP)^1^,^2^, and narrow (100-km radius) plume tails that remain in the mantle after the plume head spreads below the lithosphere^1^,^3^,^4^. Models of thermal plumes considering major phase transformations in the mantle transition zone and a heterogeneous viscosity structure in the upper mantle (see Methods) do not change these predictions radically (Supplementary Fig. 1). However, there is growing evidence that neither kilometre-scale uplifts nor narrow tails characterize starting plumes^5^,^6^,^7^,^8^,^9^,^10^. We propose that the reason is not that the entire mantle plume concept is wrong^8^,^11^,^12^, but that in its present form, it underestimates the effect of the strong chemical heterogeneity of the mantle.

More than 30 years ago, based on isotopic and trace-element geochemical data, it was suggested that a part of the oceanic crust that subducts to the core-mantle boundary (CMB) is then entrained in mantle plumes and transported back to the surface^13^; later this idea was confirmed by numerical models^14^,^15^. New geochemical data on olivine compositions of ocean island basalts and LIP magmas^16^,^17^,^18^ indicate that the fraction of recycled oceanic crust in their sources may be higher than 10%. At sublithospheric depths, basaltic oceanic crust is transformed to eclogite, which has a higher density than average mantle peridotite through most of the Earth's mantle^19^,^20^,^21^. Therefore, the entrainment of a considerable amount of eclogitic material significantly increases the plume density to produce what we will call low-buoyancy plumes (LBPs). More specifically, we only include plumes in this definition whose buoyancy in the upper mantle is reduced at least by half compared with a purely thermal plume. One example is the Siberian plume—its eclogite fraction of 10–20%, together with an excess temperature of ∼250 K, made it almost neutrally buoyant, which explains the absence of pronounced premagmatic uplift during the emplacement of the Siberian LIP^18^. Given that a large number of plumes may contain a high content of recycled crust^17^, calculations solely based on excess temperature may strongly overestimate the plume buoyancy.

Geodynamic models that feature thermochemical plumes showed a large diversity of plume behaviours^22^,^23^,^24^,^25^,^26^. These previous studies identified that the competition between thermally and chemically induced density variations, and their depth dependence, play a key role in plume dynamics. However, to advance our current understanding of plumes occurring in the Earth's mantle, these models need to incorporate additional insights from observational and experimental data. This will give us the opportunity to answer the crucial, but still open questions how LBPs can rise through the Earth's entire mantle (if at all) and which conditions favour their ascent. Here we address these questions using numerical thermomechanical modelling. The novelty of this study is that the material properties of the plume and the ambient mantle in our model are more Earth-like than what has previously been published. These properties, such as plume composition, excess temperature in the upper mantle, density and thermal expansivity, were constrained using geochemical and petrological observations and experimental data. Parameters that are less well constrained by the surface observations, such as the plume initial temperature, its volume and the mantle temperature profile, were systematically varied to investigate their influence on the plume morphology and its surface manifestations.

We find that the conditions for an LBP to ascend through the entire mantle and to cause only a negligible surface uplift on reaching the lithosphere include high plume volume together with moderate lower-mantle subadiabaticity or plume formation several hundred kilometres above the CMB. This, together with a sufficiently high temperature, allows LBPs to directly advance to the base of the lithosphere, while plumes with slightly lower buoyancy pond in a depth of 300–400 km and form pools or a second layer of hot material. We also show that the bulky tails of large and hot LBPs are stable for several tens of millions of years and that their shapes fit seismic tomography data much better than the narrow tails of thermal plumes.

## Results

### Effects of realistic buoyancy on plume rise and surface uplift

**Figure 1:**
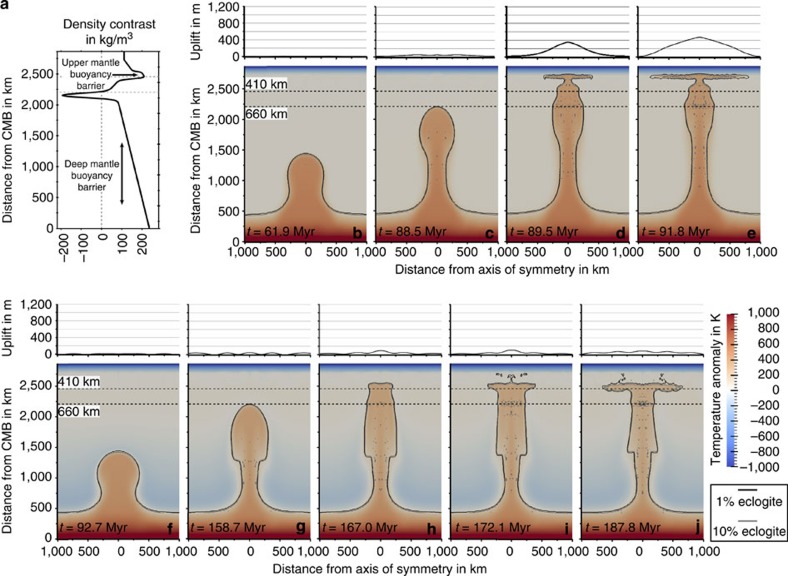
Potential barriers for the plume ascent. (**a**) Difference between the densities of eclogite and peridotite as function of depth, based on experimental data^19^,^20^,^21^. (**b**–**e**) Evolution of a ‘primary' thermochemical plume in an adiabatic mantle with an initial excess temperature of 550 K and a ‘small' initial volume of 1.22 × 10^8^ km^3^. Colours give the temperature anomaly (deviation from adiabatic temperature of the ambient mantle) and solid lines denote the composition. The related surface uplift is shown above each model section. Because of the high excess temperature, the plume buoyancy is high enough to overcome the lower mantle buoyancy barrier (**b**,**c**) and the plume reaches the upper mantle, spreads below the lithosphere (**d**,**e**) and causes a premagmatic surface uplift of 400 m. However, the maximum excess temperature of more than 350 K seems to be higher than what is typically observed^28^. (**f**–**j**) Evolution of a ‘failing' thermochemical plume in a subadiabatic mantle with an initial excess temperature of 450 K and a ‘small' initial volume of 1.22 × 10^8^ km^3^, colour scale as in (**b**–**e**). Because of the subadiabatic mantle temperature, the plume buoyancy is high enough to overcome the lower mantle buoyancy barrier (**f**,**g**) and the plume reaches the upper mantle (**h**). However, due to its low-buoyancy flux, the plume cannot cross the upper mantle buoyancy barrier (**i**) and is ponding in a depth of 300–400 km (**j**).

Our models assume an axisymmetric geometry^27^ and consider mantle compressibility, strongly temperature- and depth-dependent viscosity and major phase transformations in the mantle transition zone. Experimental data^19^,^20^,^21^ provides the depth-dependent density difference between peridotite and eclogite (Fig. 1a). Starting plumes are considered to consist of peridotite and a prescribed amount of eclogite; the mantle is modelled as purely peridotitic. More details of the modelling technique are presented in the Methods and Supplementary Fig. 2.

**Figure 2:**
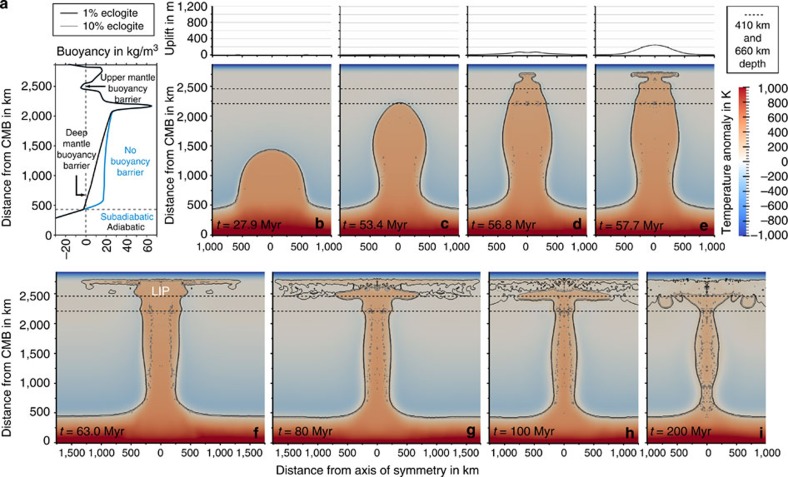
Ascent dynamics of an LBP. (**a**) Buoyancy as a function of depth for a thermochemical plume containing 15% of eclogite with an initial excess temperature of 450 K in an adiabatic (black line) and subadiabatic (blue line) mantle. The vertical dashed line marks zero buoyancy and the horizontal dashed line marks the top of the thermochemical boundary layer in the lowermost mantle. (**b**–**i**). Evolution of a thermochemical plume in a subadiabatic mantle with an initial excess temperature of 450 K and an initial volume of 3.71 × 10^8^ km^3^, that is, best fit model for the Siberian LIP. Colours give the temperature anomaly (deviation from the adiabatic mantle temperature) and solid lines denote the composition. The lines almost merge at the margin of the plume indicating a high compositional gradient. The related surface uplift is shown above each model section.

Two regions of greatest density contrast between peridotite and eclogite may act as barriers to the ascent of an eclogite-rich plume: the deep lower mantle and the upper mantle between 300 and 400 km depth (Fig. 2a, Supplementary Fig. 3). The upper mantle barrier is caused by phase transformations of pyroxenes to garnet structures and the transformation of coesite to stishovite. This leads to an increase in eclogite density, which is not compensated by density increase in peridotite until a depth of 410 km, where olivine transforms to spinel. To cross these barriers, an eclogite-rich plume needs a high excess temperature. More precisely, in an adiabatic mantle and for an initial eclogite content of 15%, a minimum excess temperature of 550 K in the lower mantle is required (see time snapshots of such a plume evolution in Fig. 1b–e). Plumes with the same eclogite content and lower excess temperatures do not reach the uppermost mantle and are ponded either in the deep lower mantle or in the mantle transition zone. Owing to adiabatic cooling and loss of heat to surroundings, the maximum excess temperature in the part of the plume that spreads below the lithosphere (Fig. 1d,e) is reduced to 370 K. This part has an average eclogite content of about 14%, and the lithosphere above the plume is uplifted by about 400 m. While the predicted surface uplift for this thermochemical plume is smaller than for a thermal plume, it still remains significant, and the excess temperature of 370 K seems to be unusually high^28^.

### Effect of mantle subadiabaticity and plume volume

To allow a cooler thermochemical plume to cross the deep mantle buoyancy barrier requires a subadiabatic temperature (a few 100 K colder than adiabatic temperature) in the deep lower mantle, as suggested by mantle convection models^29^. A plume with the same temperature has a higher temperature contrast to its surroundings and therefore higher thermal buoyancy in the subadiabatic lower mantle than in the adiabatic or in a hotter than adiabatic (that is, super-adiabatic) mantle and as a result is able to carry a larger amount of eclogite. However, large intervals of subadiabatic temperatures are likely present only in the deep lower mantle^29^ and therefore cannot help plumes to cross the upper mantle buoyancy barrier. A relatively small LBP initiated in the subadiabatic lower mantle may successfully reach the upper mantle buoyancy barrier, but then is ponded in the mantle transition zone (Fig. 1f–j). The way to overcome this obstacle is to increase the plume volume. A sufficiently large LBP starting in the subadiabatic lower mantle crosses both barriers and spreads below the lithosphere (Fig. 2). Two factors are responsible for this effect: first, the larger plume rises faster and cools less and therefore approaches the barrier with higher thermal buoyancy. Second, the larger plume volume allows deeper parts of the plume, where eclogite has a lower density than peridotite (for example, just below the 660 km discontinuity, Fig. 2a), to compensate the negative buoyancy of the barrier. In this way, the upper mantle buoyancy barrier works like a low-pass filter in seismology, allowing only large LBPs (like long-wavelength seismic waves) to pass through.

### Types of low-buoyancy thermochemical plumes

**Figure 3:**
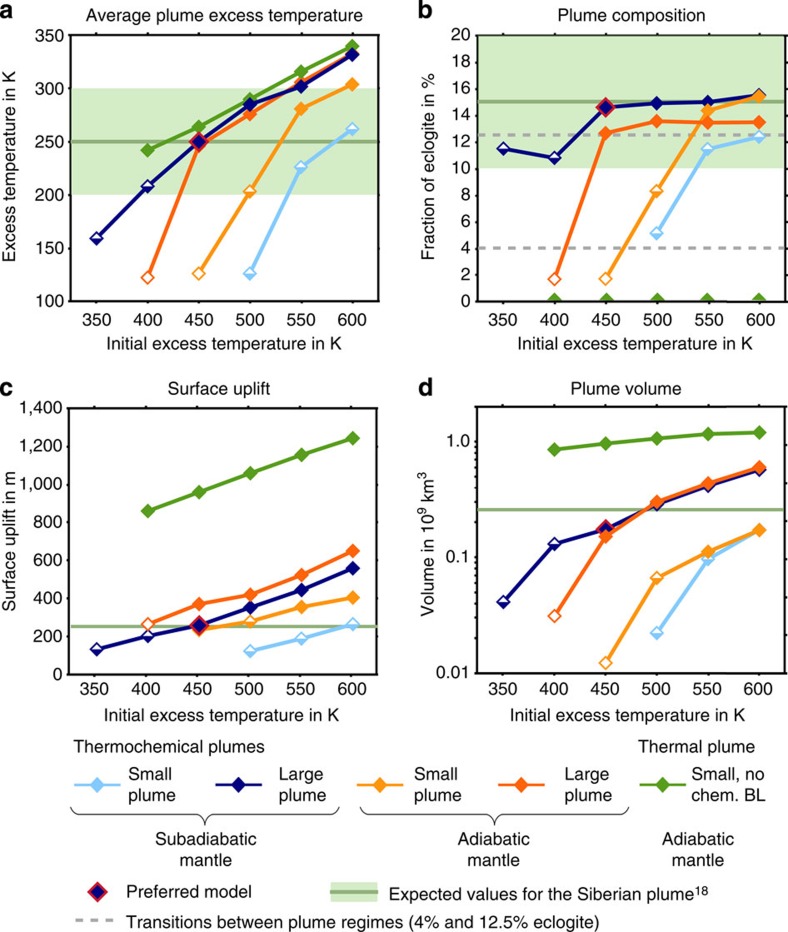
Conditions for the ascent of a LBP. Shown are the plume temperature (**a**), composition (**b**), premagmatic surface uplift (**c**) and volume (**d**) versus plume initial excess temperature for different plume initial sizes and mantle temperature profiles, leading to different dynamic regimes. Colours give the model configuration; symbols give the plume dynamic regime: primary plume (full diamonds), secondary plume from the upper mantle (half-full diamonds), secondary plume from the deep lower mantle (hollow diamonds) and failing plume (no symbol). ‘Small' plumes have an initial volume of 1.22 × 10^8^ km^3^ and ‘large' plumes 3.71 × 10^8^ km^3^. Plume excess temperature, volume and composition after arrival at the lithosphere were calculated by averaging over the area with excess temperatures higher than 100 K in a depth <300 km. Average plume excess temperature, average eclogite fraction and maximum surface uplift are presented at 1 Myr and plume volume at 5 Myr after the plumes reach the base of the lithosphere.

**Figure 4:**
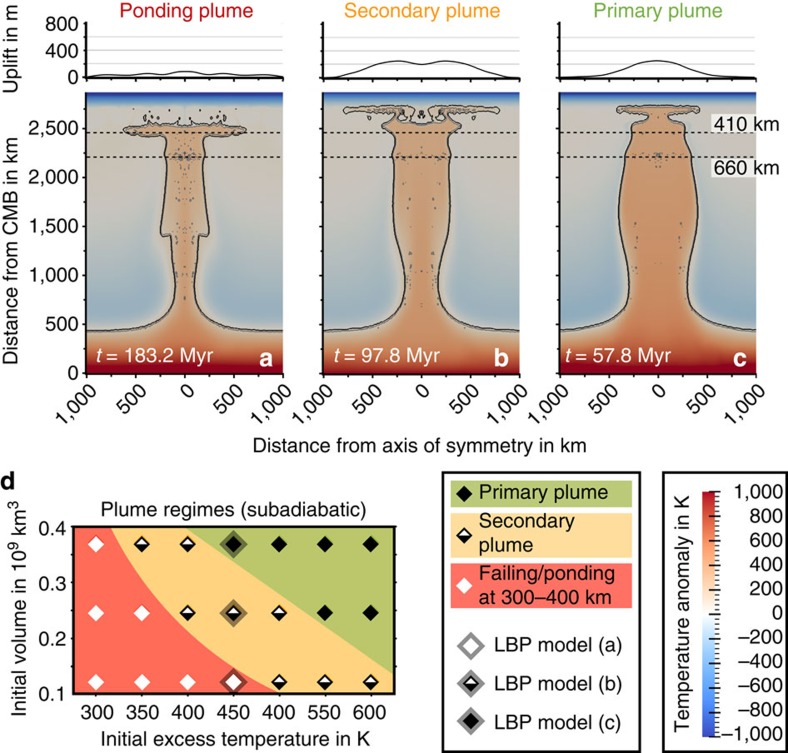
Influence of the plume volume on the plume regime for the thermochemical plumes rising in a subadiabatic mantle. Colour scale for the time snapshots as in Fig. 1. (**a**) Time snapshot of a ‘small' plume (initial volume of 1.22 × 10^8^ km^3^). The plume takes almost 200 Myr to reach the upper mantle and is not able to cross the upper-mantle buoyancy barrier. (**b**) Time snapshot of an ‘intermediate' plume (initial volume of 2.47 × 10^8^ km^3^). The plume cannot cross the upper-mantle buoyancy barrier as a whole, only secondary plumes rise from there. (**c**) Time snapshot of a ‘large' plume (initial volume of 3.71 × 10^8^ km^3^). The buoyancy flux is sufficiently large for the plume to directly advance to the base of the lithosphere (primary plume). (**d**) Plume regime in dependence of initial plume temperature and volume. Symbols and their colours are described in the figure inset.

A series of models with different plume temperatures and volumes, and adiabatic and subadiabatic mantle temperatures, shows that plumes reaching the base of the lithosphere can be categorized into three different types (Fig. 3). (1) Primary plumes that rise directly from the CMB (Fig. 4c, solid diamonds in Fig. 3). (2) Secondary plumes from the deep lower mantle (open diamonds in Fig. 3) and (3) secondary plumes from the upper mantle (Fig. 4b, half open diamonds in Fig. 3). Primary plumes have high average excess temperatures in the upper mantle of more than 200 K (Fig. 3a) and in this respect are similar to the classical plume models and plume regimes described in previous modelling studies^22^,^25^. They can transport a large fraction of eclogite from 12% up to 17% (Fig. 3b), and are supplied with new material through the plume conduit for several million years. Although they cross the upper-mantle buoyancy barrier, a portion remains in a ‘pool' at 300–400 km depth (Fig. 2f,g). A similar structure has been observed in seismic tomography models of the mantle beneath the Hawaiian islands^9^ and also has been proposed in a recent modelling study^30^. The volumes of primary plume heads approaching the base of the lithosphere can exceed 10^8^ km^3^ (Fig. 3d).

Smaller initial temperatures lead to negative buoyancy of LBPs, which causes them to pond either in the deep lower mantle, if temperatures in the lower mantle are adiabatic or super-adiabatic, or below the buoyancy barrier in the upper mantle, if the lower mantle is subadiabatic. In both cases, these ponding plumes heat the mantle above and can generate secondary plumes. The deep-rooted secondary plumes rise from the top of the thermochemical boundary layer at the CMB and are similar to what is predicted in a previous modelling study^22^. They contain only a small amount of eclogite, and have relatively low excess temperatures (<150 K) and small volumes. Secondary plumes rooted in the upper mantle form above the thermochemical plumes ponding in a depth of 300–400 km (Fig. 4a,b). These secondary plumes show various excess temperatures and eclogite contents of 4–12.5% (Fig. 3a,b) depending on the initial plume temperature, composition and volume and on the time the material ponds in this pool. However, all secondary plumes are short-living features; in all our models, due to the low volume and mixing in the asthenosphere the plume excess temperature decreases by about 10% and the fraction of eclogite by 25% in only five million years.

**Figure 5:**
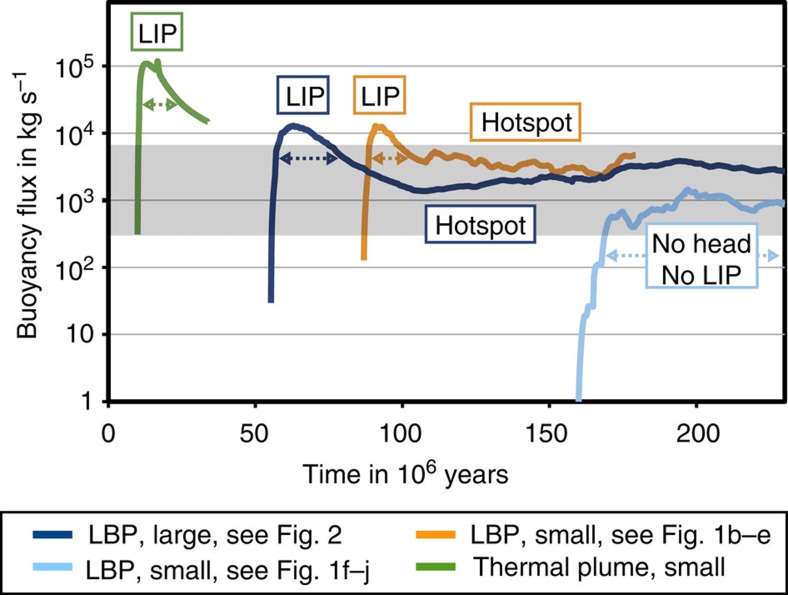
Buoyancy flux evolution of thermochemical plumes. Shown is the buoyancy flux within the plume (taken as the region with an excess temperature higher than 25 K) at a depth of 200 km. Model parameters and colouring as in Fig. 3. The three primary plumes shown (dark blue, yellow and green line) show a peak in their buoyancy flux associated with massive melting and the generation of a LIP when the plume head reaches the base of the lithosphere. They exceed the values observed for present-day hotspots^31^ denoted by the grey field, with the thermal plume (green line) reaching a significantly higher buoyancy flux than the thermochemical plumes. Subsequently, the buoyancy flux decreases to a stable level supported by the plume tail with fluctuations caused by smaller scale upwellings, representing long-time hotspot activity. The small ‘failing' thermochemical plume (light blue line) shows a much lower buoyancy flux without a pronounced peak, resulting from no plume head reaching the base of the lithosphere. The buoyancy fluxes of the tails of the presented thermochemical plumes are all within the estimates for present-day hot spots^31^.

Another property that distinguishes the different plume regimes is the associated plume buoyancy flux: the buoyancy fluxes of primary plumes show a characteristic evolution pattern with a peak in the beginning, associated with the generation of a LIP when the plume head reaches the lithosphere, and a more stable lower level flux resembling the ongoing hotspot activity caused by the plume tail (Fig. 5). As the heads of secondary plumes do not reach the base of the lithosphere and do not generate LIPs, their buoyancy flux only reflects the lower activity associated with a hotspot (Fig. 5). Note that the buoyancy fluxes associated with the plume tails of LPBs are within the range of estimates for present-day hot spots^31^ (grey field in Fig. 5).

### Conditions for LBPs to reach the lithosphere

Although all these different plume regimes might occur in the terrestrial mantle, only the primary plumes have sufficient temperatures and volumes to generate LIPs. To reach realistic excess temperatures between 200 and 300 K (ref. 28) in the upper mantle, the plume excess temperature in the deep lower mantle needs to be in a range of 400–550 K. Plumes starting in a subadiabatic mantle can ascend at lower initial temperatures than plumes rising from an adiabatic lower mantle and they can carry a higher fraction of eclogite (more than 14%, Fig. 3b) to the base of the lithosphere. The combination of high eclogite content and low-temperature results in reduced buoyancy and a smaller surface uplift (Fig. 3c). The best fit to the parameters of the Siberian plume^18^, shown by horizontal solid lines in Fig. 3, is achieved by the model of a large plume with an initial excess temperature of 450 K, carrying 15% of eclogite and rising in a subadiabatic lower mantle. When this plume arrives at the lithosphere after an ascent time of 58 Myr (Fig. 4c), which is several times longer than for a purely thermal plume, it has an average excess temperature of about 250 K and still contains 15% of eclogite. As a result, it generates a surface uplift of only 260 m, which is less than a quarter of the uplift above a purely thermal plume with the same excess temperature. The plume head volume at the base of the lithosphere amounts to 180 million km^3^ and hence is sufficient for causing massive volcanism^18^. Note that a LBP with a smaller volume under otherwise identical conditions cannot cross the buoyancy barrier at 300–400 km (Fig. 4d) and is stuck below it completely (Fig. 4a) or partially (Fig. 4b).

## Discussion

Therefore, our modelling shows that a LBP with parameters similar to the Siberian plume can indeed rise through the entire mantle, if its volume is sufficiently large and the lower mantle is subadiabatic. A possible alternative mechanism for overcoming the lower-mantle buoyancy barrier, worth further investigations, is the plume ascent from the upper boundary of thermochemical piles, which have been proposed to be present in the lowermost mantle with parts extending up to more than 1,000 km above the CMB^32^. This would allow plumes to rise from a shallower depth, where their buoyancy is positive, especially at the edges of the piles, where the surrounding mantle temperature is expected to be adiabatic.

Considering a depth-dependent plume buoyancy constrained by mineral physics and geochemical data also reveals different barriers for the ascent of LBPs compared with what was reported in previous studies^24^,^26^: Because of the negative chemical density contrast in 660–750 km depth (Fig. 1a), eclogite-bearing plumes cross the spinel-perovskite phase transition despite its negative Clapeyron slope. In addition, the employed experimental data on eclogite density^21^ indicate a buoyancy minimum close to the CMB and not in the upper part of the lower mantle, excluding stagnating plumes predicted in the lower mantle^26^.

We also note that the shapes of LBPs are different from those inferred for classical thermal plumes in the lower mantle. The plume heads are much less pronounced, even at the stage when LIPs are generated (compare Fig. 2f and Supplementary Fig. 1). LBPs also have columnar ‘tails' with a diameter of more than 500 km, which remain stable in the mantle for tens or hundreds of millions of years after the plume arrives at the base of the lithosphere (Fig. 2), in agreement with previous modelling results^22^,^23^. Hence, it should be much easier to resolve these structures with seismic methods than classical narrow plume tails^33^. Indeed, seismic tomographic observations^10^ suggest that many major plumes are broad features that can be well imaged in many models, with comparable confidence as slabs^33^. This implies that many major plumes in fact are thermochemical rather than purely thermal, and are described better by LBP models than by classical models. This conclusion is in accordance with the growing geochemical evidence that mantle sources of both ocean island basalts and LIPs have heterogeneous compositions^17^.

If many major mantle plumes are LBPs, the entire plume concept will need to be reconsidered. While an LBP requires more than 50 Myr for its ascent, a purely thermal plume reaches the lithosphere in less than 20 Myr, the consequence being that the plume buoyancy fluxes of LBPs are radically different, in particular during the LIP stage, where the buoyancy flux of thermal plumes is one order of magnitude higher (Fig. 5). Moreover, after the rise of a LBP to the lithosphere and the generation of the associated LIP, in contrast to the classical plume theory, large-scale thermal heterogeneities remain in the mantle for a long time and may act as ascent channels for new hot mantle material tens or even hundred million years later. This stable root of the LBP supports the longevity of the hot-spot stage of the plume evolution and involves a buoyancy flux well within the range of estimates for present-day hot spots (Fig. 5).

In summary, the consideration of thermochemical plumes with high content of eclogite constrained by geochemical data together with a realistic depth-dependent density contrast between eclogite and peridotite as well as lower mantle subadiabaticity successfully reproduces the probable characteristics of the sources of LIPs and resolves the controversy of the classical mantle plume concept concerning high premagmatic uplifts and mantle plume shapes.

## Methods

### Model set-up

The two-dimensional axisymmetric version of the Citcom code^34^ we use here^27^ employs the anelastic liquid approximation and treats the mantle as compressible^35^. The model domain comprises the whole mantle in vertical direction and extends over a distance of 2,870 km horizontally. We compute models with both adiabatic and subadiabatic geotherms (Supplementary Fig. 2a). Here we do not aim to study the very complex processes of the formation of a thermochemical bottom boundary layer and the entrainment of a dense phase into the mantle plume^22^,^23^,^36^,^37^,^38^,^39^,^40^,^41^,^42^,^43^,^44^. Instead, we take a simplified approach assuming that such a layer already exists and contains a prescribed amount of the eclogitic material (Supplementary Fig. 2b). To start the plume ascent, we also assume that there is an initial temperature perturbation at the central axis that previously entrained a certain fraction of eclogite. The plume initial temperature and volume were varied by changing the excess temperature (relative to the adiabatic temperature) of this perturbation between 300 and 600 K and its volume between 1.22 × 10^8^ and 3.71 × 10^8^ km^3^. The amount of eclogite within the thermally perturbed region (initial plume) was assumed to be 15%, as estimated for the Siberian plume^18^.

Different phase transitions are incorporated for the two chemical components resulting in a depth-dependent density contrast between peridotite and eclogite, which is based on recent experimental data^19^,^20^,^21^ and also includes the effect of composition-dependent compressibility in the lower mantle (Supplementary Fig. 2c,d). We use a temperature- and depth-dependent viscosity modified from a previous modelling study^27^ (Supplementary Fig. 2e) and additionally test the effect of increasing the activation energy by a factor of 3. The depth-dependent thermal expansivity is based on mineral physics data^45^ (Supplementary Fig. 2f). Together with the depth-dependent compositional density contrast and the plume temperature changes due to adiabatic cooling, this results in a typical buoyancy profile for a rising plume (Supplementary Fig. 3a) constrained by geochemical observations and experimental data, which is one of the novel aspects of this work. Previous studies did not focus on conditions for the ascent of eclogite-bearing mantle plumes through the whole mantle and thus either used a simplified^22^,^23^ or constant^24^,^25^ chemical density contrast, a constant thermal expansivity^22^,^23^,^25^,^26^ or investigated only the upper^30^ or only the lower^26^ mantle.

All parameters used in the model are presented in Table 1.

Note that increasing the temperature dependence of viscosity changes the ascent time of LBPs, but does not significantly influence the plume shape, dynamics or surface manifestations (Supplementary Figs 4 and 5).

### Numerical technique

Using the anelastic liquid approximation and treating the mantle as compressible^35^ leads to the following equations


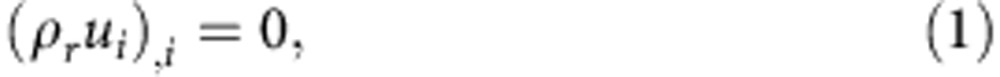



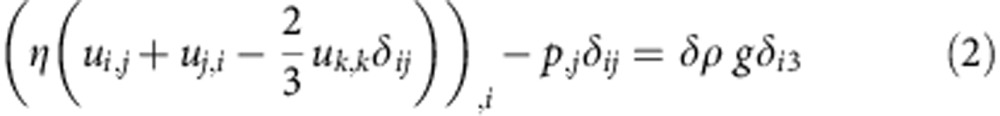


with the density anomaly 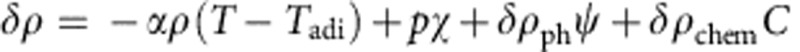, and


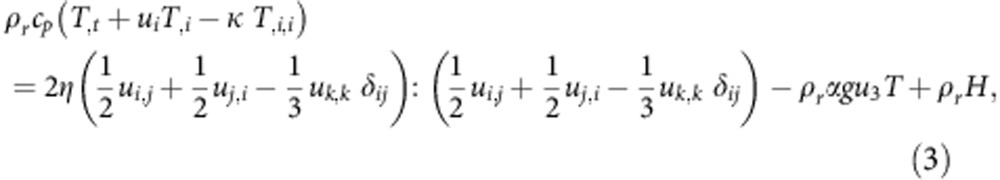


which are solved non-dimensionalized and in a cylindrical coordinate system^36^. *ρ*_*r*_ is the radial density profile, **u** is the velocity, *η* is the viscosity, *p* is the dynamic pressure, *g* is the gravitational acceleration, *α* is the thermal expansivity, *T*_adi_ is the adiabatic temperature, *χ* is the compressibility, *δρ*_ph_ is the density change across a phase boundary, *ψ* is the phase function, *δρ*_chem_ is the depth-dependent chemical density contrast between eclogite and peridotite, *C* is the fraction of eclogite, *c*_*p*_ is the specific heat capacity, *κ* is the thermal diffusivity and *H* is the specific radiogenic heat production rate. Values of all material parameters are given in Table 1.

We use the Adams–Williamson Equation of state, which leads to a radial density profile of


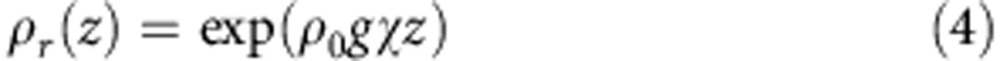


with the reference density *ρ*_0_.

The depth dependence of viscosity *η*_*r*_(*z*) is given in Supplementary Fig. 2e, and the temperature dependence^27^ is


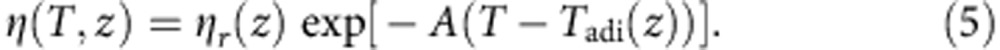


The adiabatic temperature profile *T*_adi_ is computed during the initialization of the model by solving equation


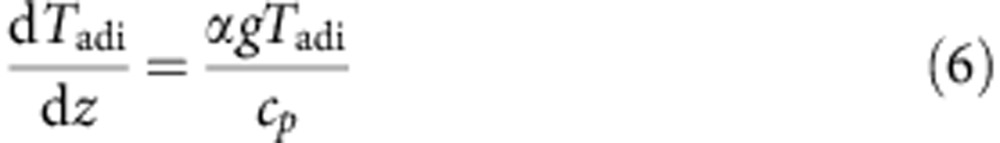


iteratively, starting from the given surface temperature *T*_S_.

Citcom uses finite elements, and a multigrid solver using an Uzawa algorithm is incorporated to solve the coupled momentum and mass conservation equation. The resulting velocity is employed to solve the energy equation with a streamline upwind the Petrov–Galerkin scheme. The compositional field is transported by markers and interpolated onto the nodes with a tracer ratio method. A predictor–corrector scheme is used for updating the marker positions.

The surface uplift is calculated using the normal stresses acting on the upper surface:


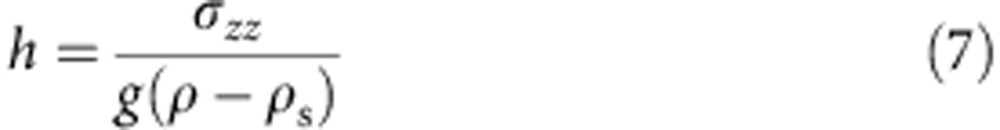


with 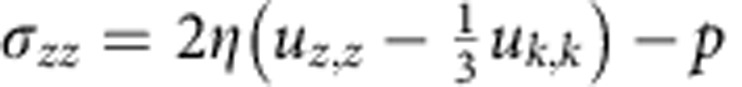 and *ρ*_s_ being the density contrast at the surface, which is 1 kg m^−3^ where *h*<0 (water) and 0 where *h*>0 (air). A feature to note is the self-adaptive coordinate transformation integration scheme that increases the accuracy of the calculated surface topography near the axis of symmetry^27^.

The plume buoyancy fluxes *B* are calculated at 200 km depth, integrated over the area with excess temperatures exceeding 25 K and using the following relation:


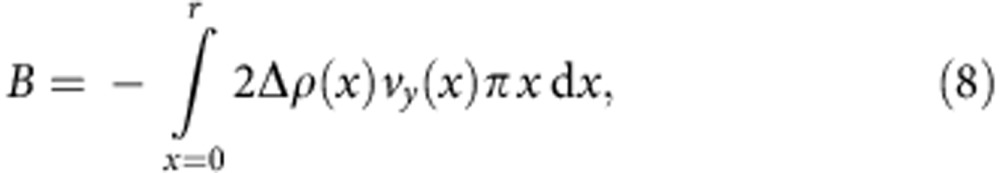


with Δ*ρ* being the density difference between the plume and an undisturbed mantle, including effects of both temperature and composition, *x* the radial direction and *y* the vertical direction.

All changes made to the original version of the code presented in a previous study^27^ are available from the authors on request.

### Model approximations

One assumption we make is that the length scale of chemical heterogeneities within the plume is so small that dense eclogite bodies do not sink in peridotitic material. Estimates based on the Stokes relation for the velocity of a sinking sphere show that for a higher plume viscosity than 10^17^ Pa s this assumption holds if the radii of the spherical eclogite bodies are smaller than 100 m.

Note also that we do not include plume–lithosphere interaction or non-linear viscosities in our model. Because of that, any predictions exceeding the first few million years after the plume arrival at the base of the lithosphere and related to mixing of material in the mantle are not as precise as our results regarding the ascent of the plume, which is the focus of this study. In addition, considering these processes strongly accelerates the development and spreading of the plume head in the upper mantle (as demonstrated in two-dimensional Cartesian models^18^), causing a higher and shorter peak in the buoyancy flux associated with the LIP than what our models show (Fig. 5).

## Author contributions

Both authors jointly designed the models, interpreted modelling results and wrote the paper; J.D. set up and performed the computations.

## Additional information

**How to cite this article:** Juliane Dannberg & Stephan V. Sobolev Low-buoyancy thermochemical plumes resolve controversy of classical mantle plume concept. *Nat. Commun.* 6:6960 doi: 10.1038/ncomms7960 (2015).

## Supplementary Material

Supplementary Information Supplementary Figures 1-5

## Figures and Tables

**Table 1:** Values of physical parameters and constants.

**Parameters with physical units**	
Earth's radius	6.371 × 10^6^ m
Mantle thickness	2.870 × 10^6^ m
Initial volume (small plume)	1.22 × 10^8^ km^3^
Initial volume (intermediate plume)	2.47 × 10^8^ km^3^
Initial volume (large plume)	3.71 × 10^8^ km^3^
Thickness of the bottom thermochemical BL (thermochemical models)	430 km
Thickness of the bottom thermochemical BL (thermal models)	100 km
Temperature difference surface—CMB Δ*T*	3,500 K
surface temperature *T*_S_	273 K
Temperature increase across the top thermal boundary layer	1,220 K
Temperature increase across the bottom thermal boundary layer	1,200 K
Surface density *ρ*_0_	3,400 kg m^−3^
Reference viscosity *η*_0_	8.44 × 10^21^ Pa s
Gravitational acceleration *g*	10 m s^−2^
Thermal diffusivity (surface) *κ*_0_*	7 × 10^−7 ^m^2^ s^−1^
Thermal expansivity (surface) *α*_0_	4.2 × 10^−5^ K^−1^
Specific heat *c*_*p*_	1,000 J kg^−1^ K^−1^
Radiogenic heat production rate *H*	5.9 × 10^−12^ W kg^−1^
Mantle compressibility *χ*†	5.124 10^−12^ Pa^−1^
Clapeyron slope of the 410-km phase transition *γ*_410_	1 MPa K^−1^
Clapeyron slope of the 660-km phase transition *γ*_660_	−1 MPa K^−1^
Prefactor in the temperature dependence of viscosity *A*‡	3.9473 × 10^−3^resp.1.3 × 10^−2^

CMB, core-mantle boundary.

^*^The thermal diffusivity increases linearly from the surface to the core-mantle boundary by a factor of 2.18 (from ref. 27).

^†^We use the Adams–Williamson equation of state, resulting in a depth-dependent density in the form of *ρ*(*z*)=exp(*ρ*_0_*g χz*). Density changes caused by phase transitions are applied additionally.

^‡^We use a viscosity law^27^ in the form of *η*(*T*,*z*)=*η*_*r*_(*z*) exp(−*A*(*T*-*T*_adi_(*z*))), with *η*(*z*) for the average mantle temperature being the viscosity profile shown in Supplementary Fig. 1(e). *A*=3.9473 × 10^−3^ (as in ref. 27) corresponds to a viscosity range of six orders of magnitude for Δ*T*=3,500 K (temperature difference surface—CMB). To examine the effect of a higher temperature dependence of viscosity on plume dynamics, we also performed computations with a three times higher activation energy (that is, *A*=1.3 × 10^−2^).
