# Supplementary material for: Low-buoyancy thermochemical plumes resolve controversy of classical mantle plume concept
Source: Nat Commun. 2015 Apr 24;6:6960. doi: 10.1038/ncomms7960 (PMC4421820; doi:10.1038/ncomms7960)
Supplement: Supplementary Information — Supplementary Figures 1-5 [file ncomms7960-s1.pdf]

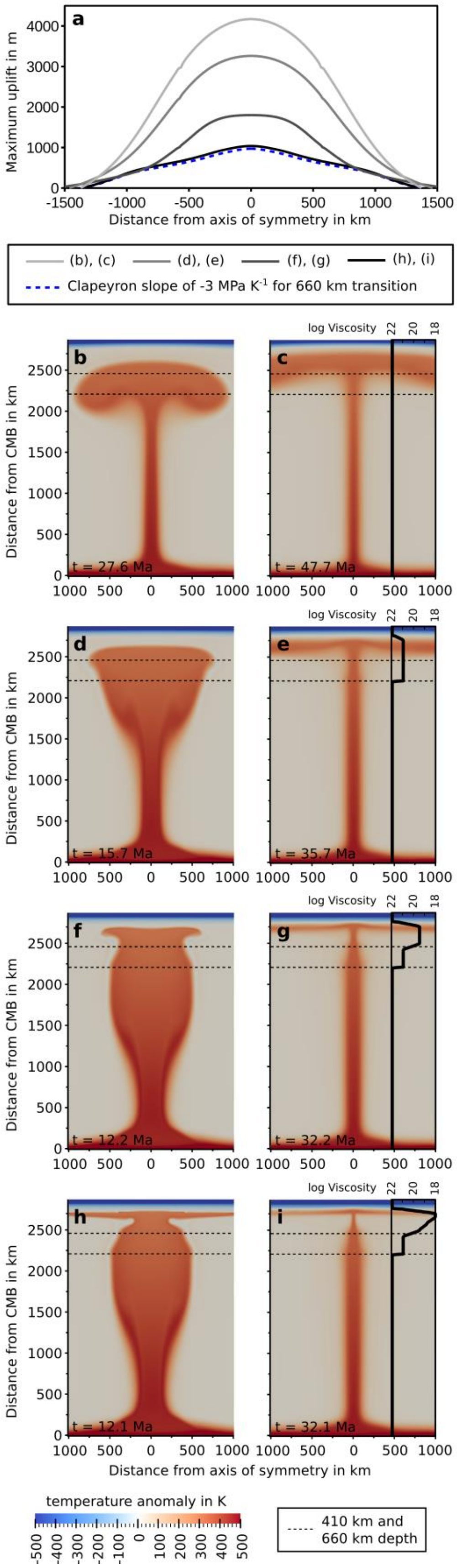

Supplementary Figure 1. Axisymmetric models of thermal mantle plumes and associated surface uplifts. (a) Maximal surface uplift versus distance above the spreading plume head computed for different viscosity structures of the upper mantle. All models include major olivine phase transitions. The dashed blue line shows the surface uplift obtained in a model with the viscosity profile of (h,i) and the highly negative Clapeyron slope of the 660 km phase transition used in a previous study[27]. This is the minimum surface uplift that is expected for a thermal plume of this size and temperature. (b, c) Time snapshots of plume excess temperature for the plume rising in a homogeneous mantle. The viscosity model is shown in the inset in the right panel. (d, e) Time snapshots of plume excess temperature for the plume rising in a mantle with reduced viscosity in the upper mantle and transition zone. (f, g) The same model as in (d, e), but with a low-viscosity upper mantle. (h, i) The same model as in (f, g), but with an additional low-viscosity layer in the asthenosphere. All plumes in (a)-(g) have an initial radius of 840 km, an initial excess temperature of 450 K and rise in an adiabatic mantle.

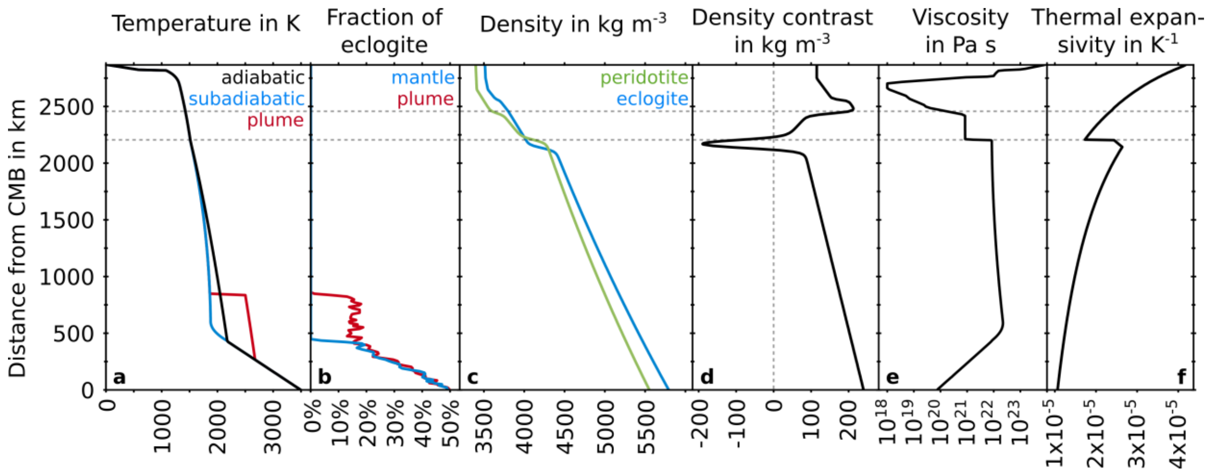

Supplementary Figure 2. Initial conditions and material model. (a) Initial temperature profile for mantle abiabaticity (black), subadiabaticity (blue) and exemplary initial temperature perturbation of the starting plume (red). (b) Representative profiles of the percentage of eclogite in the mantle (blue) and intersecting the initial temperature perturbation of the starting plume (red). The oscillations in the profiles are due to the probabilistic nature of the method of assigning initial values to the markers that are used for the transport of compositional fields. (c) Density profiles of peridotite (green) and eclogite (blue) estimated from experimental data[19-21]. (d) Density difference between peridotite and eclogite resulting from subtracting the profiles shown in (c) from each other. Positive values mark a higher eclogite density and negative values mark a higher density of peridotite. (e) Mantle viscosity profile modified from a previous study[27], including only depth-dependence; for the temperature dependence see Table 1. (f) Thermal expansivity profile derived from mineral physics data[45].

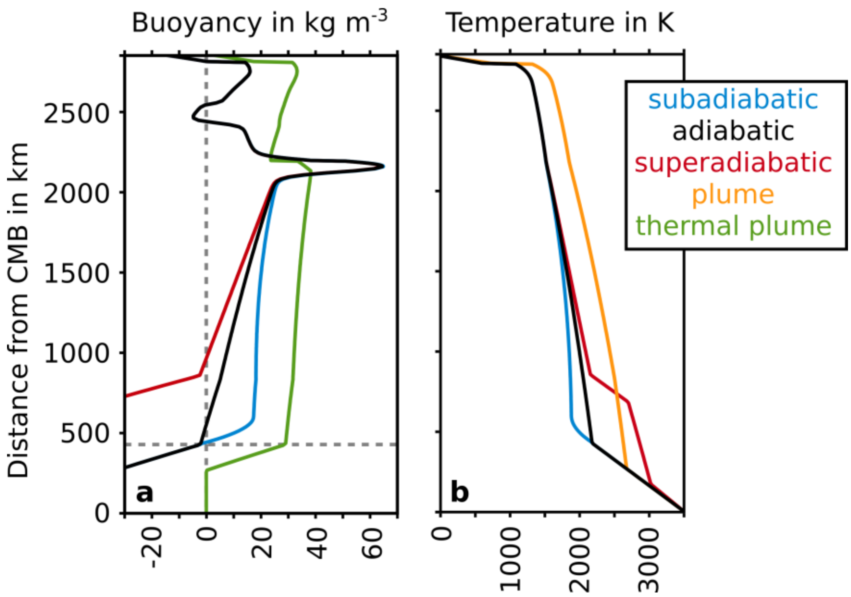

Supplementary Figure 3. (a) Buoyancy in dependence of depth for a thermo-chemical plume containing 15% of eclogite with an initial excess temperature of 450 K in an adiabatic (black line), subadiabatic (blue line) and superadiabatic (red line) mantle and for a thermal plume with an initial excess temperature of 450 K in an adiabatic mantle (green line). The vertical dashed line marks zero buoyancy and the horizontal dashed line marks the top of the thermo-chemical boundary layer in the lowermost mantle. There are two barriers for the ascent of a thermo-chemical plume: one in the lower mantle just above the boundary layer and one in the upper mantle between 300 and 400 km depth. Mantle subadiabaticity increases the plume buoyancy in the lowermost mantle and enables the plume to cross this first barrier. The profiles were calculated by adding the thermal buoyancy resulting from the temperatures in (b) and the chemical buoyancy caused by the depth-dependent density difference between peridotite and eclogite as shown in Supplementary Fig. 2d. (b) Corresponding temperature profiles for the adiabatic (black line), subadiabatic (blue line) and superadiabatic (red line) mantle as well as for a plume following an adiabat with an initial excess temperature of 450 K (yellow line, not including temperature changes due to conduction).

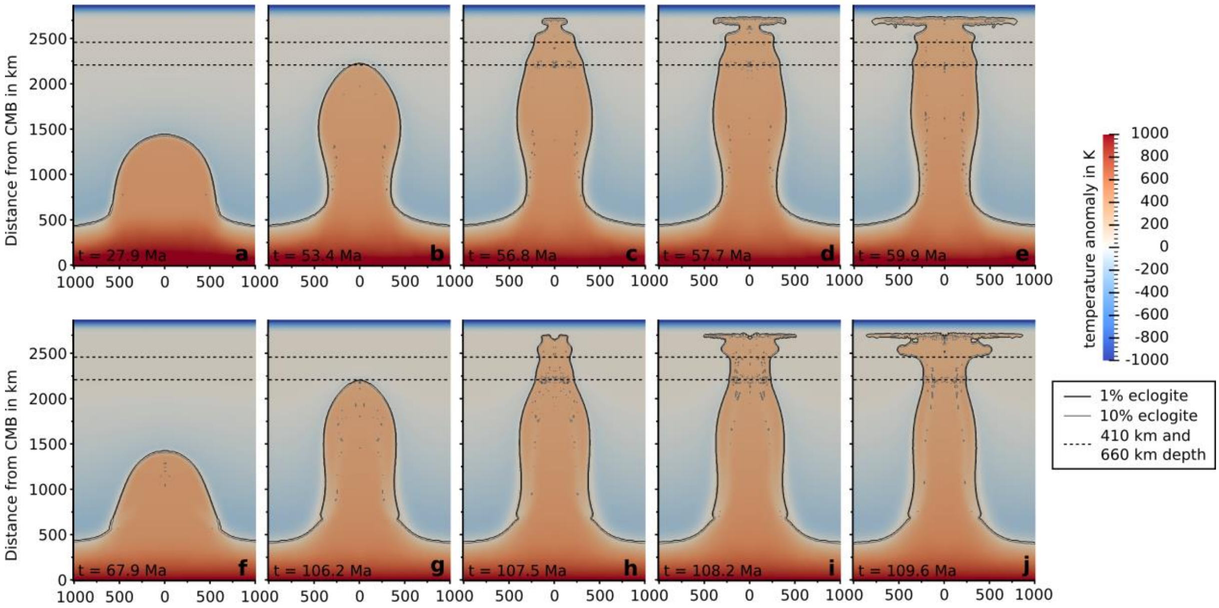

Supplementary Figure 4. Influence of strongly temperature-dependent viscosity on the dynamics of LBPs. Panels (a-e) show time snapshots of the model presented in Fig. 1 (i.e. the model that fits the Siberian Traps best), panels (f-j) show a computation with the similar setup, except for a 30 times stronger temperature dependence of viscosity, achieved by increasing the activation energy by a factor of 3, and an initial excess temperature of 425K. The comparison shows that the ascent in the lower mantle is delayed because of the increased viscosity in the low-temperature subadiabatic regions. However, there is no significant influence on the plume shape; in particular the wide plume tail in the lower mantle is present in both models.

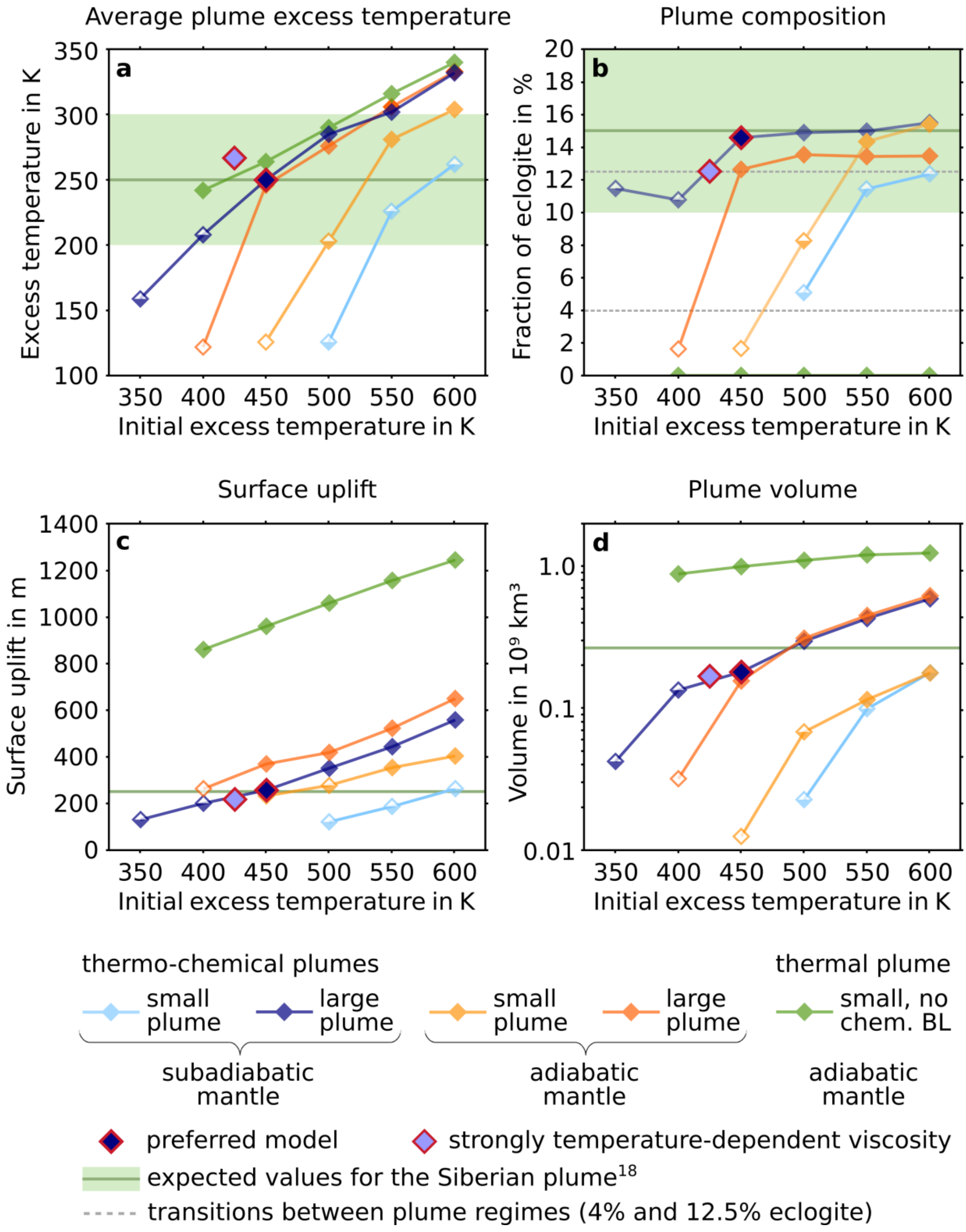

Supplementary Figure 5. Influence of temperature-dependent viscosity on the plume surface manifestations. Shown are the same models as in Fig. 3 (with identical colours and symbols), together with an additional model (violet dot) with a “large” initial volume and strongly increased temperature-dependence of viscosity (see Supplementary Fig. 4) shortly after arrival at the lithosphere, showing a similar behaviour to the preferred model.
